# ‘Guttas Campus’ - participants’ experiences of a group-based intervention to prevent school dropout

**DOI:** 10.1192/j.eurpsy.2024.1696

**Published:** 2024-08-27

**Authors:** G. Ramdal, R. Wynn

**Affiliations:** ^1^Social Education; ^2^Clinical Medicine, UiT The Arctic University of Norway, Tromsø; ^3^Department of Education, ICT and Learning, Østfold University College, Halden, Norway

## Abstract

**Introduction:**

It is important to prevent school dropout and to help students who have dropped out re-enroll in school. Dropping out of school is associated with an increased risk of unemployment, low salaries, and receiving social security or disability benefits. In this study, we interviewed participants in ‘Guttas Campus’ (The Boys’ Camp), which is a group-based intervention that aims to support disengaged boys from the 9^th^ grade and through their transition to high school. The intervention consists of a two-week learning camp. The students subsequently participate in mentoring groups, with teachers and other camp participants, for a period of 18 months.

**Objectives:**

We present a study of a school dropout prevention program.

**Methods:**

16 students were interviewed qualitatively. The interview data were analysed by drawing on the method of Grounded Theory.

**Results:**

When the students who have completed the learning camp were asked what they believed were the most important and useful parts of the intervention, some common themes emerged: 1) The learning camp community provided a safe environment and helped give the participants learning and coping experiences that increased their self-confidence. 2) The students brought up the method of teaching, which they described as more persistent, adaptive and encouraging than they had been used to from regular school. 3) The students also mentioned the intervention’s focus on character strengths such as willpower, self-control and optimism as central to increasing their motivation to learn.

**Conclusions:**

The students that were interviewed were generally positive to the intervention, as mentioned several factors that they believed were useful in increasing their motivation and ability to learn.

**Disclosure of Interest:**

None Declared

